# Integrating Stochastic and Deterministic Process in the Biogeography of N_2_-Fixing Cyanobacterium *Candidatus* Atelocyanobacterium Thalassa

**DOI:** 10.3389/fmicb.2021.654646

**Published:** 2021-10-21

**Authors:** Liuyang Li, Chao Wu, Danyue Huang, Changling Ding, Yuqiu Wei, Jun Sun

**Affiliations:** ^1^College of Marine Science and Technology, China University of Geosciences, Wuhan, China; ^2^State Key Laboratory of Microbial Metabolism, School of Life Sciences and Biotechnology, Shanghai Jiao Tong University, Shanghai, China; ^3^Research Centre for Indian Ocean Ecosystem, Tianjin University of Science and Technology, Tianjin, China; ^4^School of Oceanography, Shanghai Jiao Tong University, Shanghai, China; ^5^College of Biotechnology, Tianjin University of Science and Technology, Tianjin, China; ^6^Key Laboratory of Sustainable Development of Marine Fisheries, Ministry of Agriculture and Rural Affairs, Yellow Sea Fisheries Research Institute, Chinese Academy of Fishery Sciences, Qingdao, China

**Keywords:** cyanobacteria, *nifH*, UCYN-A, community assembly, stochasticity, determinism, temperature

## Abstract

UCYN-A is one of the most widespread and important marine diazotrophs. Its unusual distribution in both cold/warm and coastal/oceanic waters challenges current understanding about what drives the biogeography of diazotrophs. This study assessed the community assembly processes of the nitrogen-fixing cyanobacterium UCYN-A, developing a framework of assembly processes underpinning the microbial biogeography and diversity. High-throughput sequencing and a qPCR approach targeting the *nifH* gene were used to investigate three tropical seas: the Bay of Bengal, the Western Pacific Ocean, and the South China Sea. Based on the neutral community model and two types of null models calculating the β-nearest taxon index and the normalized stochasticity ratio, we found that stochastic assembly processes could explain 66–92% of the community assembly; thus, they exert overwhelming influence on UCYN-A biogeography and diversity. Among the deterministic processes, temperature and coastal/oceanic position appeared to be the principal environmental factors driving UCYN-A diversity. In addition, a close linkage between assembly processes and UCYN-A abundance/diversity/drivers can provide clues for the unusual global distribution of UCYN-A.

## Introduction

Diazotrophs play a fundamental role in biogeochemical processes by providing bioavailable nitrogen (Sohm et al., 2011; Karl et al., 2012), which determines the primary production in the ocean (Postgate, 1982; Gruber and Sarmiento, 1997; Karl et al., 1997). UCYN-A (*Candidatus* Atelocyanobacterium thalassa) is considered to be the most abundant unicellular diazotroph in the global ocean (Moisander et al., 2010; Luo et al., 2012; Martinez-Perez et al., 2016; Turk-Kubo et al., 2018; Zehr and Capone, 2020). It has been shown that UCYN-A exchanges fixed nitrogen for fixed carbon from its haptophyte host (Thompson et al., 2012; Zehr, 2015; Martinez-Perez et al., 2016). The combination of an obligate symbiosis with haptophyte algae (Thompson et al., 2012; Krupke et al., 2014, 2015; Zehr, 2015; Cabello et al., 2016; Cornejo-Castillo et al., 2016; Martinez-Perez et al., 2016; Zehr et al., 2016; Munoz-Marin et al., 2019; Zehr and Capone, 2020; Farnelid et al., 2021) and cosmopolitan distribution (Moisander et al., 2010; Farnelid et al., 2016; Harding et al., 2018; Shiozaki et al., 2018; Cabello et al., 2020; Tang et al., 2020) makes UCYN-A unusual in the world’s ocean. A recent study has suggested that N_2_ fixation, instead of nitrate (NO_3_^–^) or ammonium (NH_4_^+^) utilization, is important for the UCYN-A/haptophyte symbiosis to meet their nitrogen demands under conditions of replete dissolved inorganic nitrogen (DIN) (Mills et al., 2020). These findings expanded our knowledge of the spatial distribution and scale of habitat preference of UCYN-A, which were different from the well-known cyanobacterium *Trichodesmium* and the diatom symbiont *Richelia* that prefer warm (Breitbarth et al., 2007) and oligotrophic seawater (Tyrrell, 1999). Although UCYN-A was originally thought to be of low genetic diversity (Tripp et al., 2010), it is currently known to have formed at least four sublineages (e.g., UCYN-A1, UCYN-A2, UCYN-A3, and UCYN-A4) (Turk-Kubo et al., 2017; Henke et al., 2018), according to the nitrogenase (*nifH*) phylogeny of nucleotide sequences (Thompson et al., 2014; Farnelid et al., 2016). Additionally, different sublineages were inferred as being potentially distinct ecotypes (Turk-Kubo et al., 2017). The question of whether symbionts with such self-sufficiency and ability to survive over a wide range of temperatures were less susceptible to niche-based selection, or instead they formed versatile ecotypes to adapt to various environments owing to niche differentiation, leads us to reconsider how the biogeography and diversity of UCYN-A are assembled.

Community assembly processes are divided into two categories, namely, deterministic (niche-based) and stochastic (neutral-based) processes (Stegen et al., 2012; Wang et al., 2013; Dini-Andreote et al., 2015; Tripathi et al., 2018). Niche theory assumes that microbial communities are controlled by deterministic processes consisting of abiotic and biotic screening, owing to habitat preferences and species fitness (Fargione et al., 2003; Bahram et al., 2016). On the contrary, neutral theory supposes that ecologically equivalent members in microbial communities are shaped by stochastic processes including random birth, death, and dispersal events (Chave, 2004; Bahram et al., 2016; Zhou and Ning, 2017; Tripathi et al., 2018). These ecological processes can drive community assembly simultaneously (Fargione et al., 2003; Langenheder and Szekely, 2011; Zhou and Ning, 2017; Jiao and Lu, 2020). Furthermore, some factors are important in mediating the balance between stochastic and deterministic assembly of microbial communities, such as the pH and temperature of the soil environment (Tripathi et al., 2018; Jiao and Lu, 2020). However, it is still challenging to uncover the community assembly process and the factors mediating community determinism/stochasticity in various ecosystems (e.g., marine habitats).

Despite the increasing data available from next-generation sequencing studies of nitrogenase genes (Turk-Kubo et al., 2017; Henke et al., 2018; Cornejo-Castillo et al., 2019; Farnelid et al., 2019; Moreira-Coello et al., 2019; Cabello et al., 2020; Gradoville et al., 2020), the biogeography and diversity of UCYN-A have been analyzed mainly from a deterministic perspective, and the stochasticity in UCYN-A community assembly is still unclear. Accordingly, our limited understanding of the underlying mechanism in biogeographic variations of UCYN-A constrains our ability to incorporate them into ecological models. The Bay of Bengal (BOB) has received far less attention in research on UCYN-A than has the South China Sea (SCS) and the Western Pacific Ocean (WPO). As a crossroads of the BOB and WPO, the SCS is undoubtedly influenced by neighboring physical processes, such as ocean currents (e.g., the Kuroshio Current) (Shiozaki et al., 2013), and other physical processes including upwellings (Voss et al., 2006; Zhang et al., 2015), mesoscale eddies (Li et al., 2021a), or river plumes (Zhang et al., 2014). In particular, high temperature, stratification, and oligotrophic seawater make these waters an ideal habitat for supporting the growth of diazotrophs (Kong et al., 2011). On the other hand, UCYN-A has also been observed active in N-replete conditions (Mills et al., 2020; Tang et al., 2020). To date, however, the biogeography of UCYN-A remains patchy (Turk-Kubo et al., 2017), and there is a lack of comprehensive knowledge on the assembly mechanism of microbial communities throughout the heterogenetic BOB, WPO, and SCS.

Here, we performed ecological surveys in the BOB, WPO, and SCS using *nifH* gene-based high-throughput sequencing and qPCR. Our aims were to (I) develop a comprehensive picture of large-scale variability of UCYN-A in the tropical seas; (II) uncover the relative contribution of stochastic and deterministic processes of the UCYN-A community in tropical seas; and (III) reveal the drivers mediating the assembly processes. We hope that this study will provide a novel context for the spatial heterogeneity of UCYN-A in the BOB, WPO, and SCS, as well as providing some inspirations for researchers to explore the mechanisms underlying the unusual distribution of UCYN-A.

## Materials and Methods

### Sample Collection and Analysis of Environmental Parameters

Surface seawater samples (5 m) were collected from the BOB cruise (onboard R/V “*Dongfanghong* 2” from November to December 2016; latitude, 5–17°N), WPO cruise (onboard R/V “*Kexue*” from October to November 2017, 2–18°N), western South China Sea (wSCS), and central South China Sea (cSCS) cruises (onboard R/V “*Shiyan* 3” from September to October 2016 and “*Shiyan* 1” from March to May 2016, respectively; 10–22°N). Three samples from the cSCS cruise were strongly affected by the Pearl River plume, and they were grouped into the northern SCS region (nSCS) in subsequent analyses. Seawater samples were collected using Polyethylene (PE) buckets that were previously rinsed with 10% HCl and Milli-Q water. To obtain the material for *nifH* gene-based high-throughput sequencing and qPCR analysis, 0.5–4 L of seawater were filtered through a filter of 0.22-μm pore size (47 mm in diameter, Millipore, Eschborn, Germany) with low pressure (>−0.03 Mp). The filters were flash-frozen in liquid nitrogen on board, transported to the laboratory, and stored at −80°C until DNA extraction.

Sea surface temperature and salinity were determined and recorded using a conductivity–temperature–depth (CTD) system on board (Seabird SBE 911Plus, Sea-Bird Electronics, WA, United States). Sample analysis for other physicochemical parameters, such as inorganic nutrients and Chlorophyll *a* (Chl *a*), were consistent with our previous work (Wu et al., 2019; Li et al., 2021b). Briefly, we used 100-ml PE bottles to collect seawater for nutrient analysis. The nutrients, including nitrate (NO_3_^–^) or nitrite (NO_2_^–^), ammonium (NH^4+^), silicate (SiO_3_^2–^), and phosphate (PO_4_^3–^), were measured using the copper–cadmium column reduction methods, indophenol blue method, silico-molybdate complex methods, and phosphor-molybdate complex methods, respectively (Pai et al., 2001; Guo et al., 2014). Furthermore, we used a 0.7-μm pore size GF/F filter (25 mm diameter, Waterman, Florham Park, NJ, United States) to collect subsamples for Chl *a* analysis under a pressure higher than −10 mmHg. The filters were subsequently stored in aluminum foil paper bags at −20°C. Chl *a* was extracted with 90% acetone and measured using a Turner-Designs Trilogy^®^ fluorometer (San Jose, CA, United States) in the laboratory (Welschmeyer, 1994; Liu et al., 2015). We used the distance from the sea surface to the bottom (DSB) and salinity as indirect indicators associated with coastal/oceanic habitats.

### DNA Extraction and Sequencing

DNA were extracted and purified using DNeasy PowerWater^®^ Kit (Qiagen, Hilden, Germany) as the manufacturer’s protocol described. We used an ND-2000 Nanodrop spectrometer (Thermal Scientific, Wilmington, DE, United States) to check the quantity and quality of genomic DNA. In the nested polymerase chain reaction (nested PCR), universal *nifH* primers nifH3/nifH4 (Zehr and Turner, 2001) and UCYN-A-specific *nifH* primers with 5′ common sequence linkers (Turk-Kubo et al., 2017) were used, respectively, in the first amplification and second amplification (Supplementary Table 1). PCRs were conducted in quintuplicate with a Veriti 9902 thermocycler (Applied Biosystems, Foster City, CA, United States). The reaction system, reaction conditions, and thermocycling parameters for nested PCR analysis are described in Supplementary Tables 2, 3 in the Supporting Information. The libraries were constructed and sequenced using paired-end chemistry (PE300) on the Illumina MiseqPE300 platform (Illumina, San Diego, CA, United States).

### Quality Control and Sequencing Data Processing

The raw sequencing reads were demultiplexed and quality filtered by their barcode sequences under the limit of no more than one mismatch. The open source bioinformatics pipeline QIIME was applied to preliminarily analyze and process the *nifH* gene sequences (Caporaso et al., 2010). The paired-ended sequences were merged *via* FLASH (v1.2.7) software (Magoc and Salzberg, 2011), and the generated raw tags were quality filtered using Trimmomatic (v0.33) software (Bolger et al., 2014). After undertaking the above processes, we obtained high-quality clean tags without the barcodes, linker sequences, and primers. Chimeras were also removed to get effective tags by using UCHIME (v4.2) software (Edgar et al., 2011). Furthermore, singletons were eliminated to minimize sequencing errors. We set a 99% similarity level to cluster the remaining tags to different operational taxonomic units (OTUs) (Cornejo-Castillo et al., 2019) using software USEARCH (v10.0) (Edgar, 2010). After normalization (1,395,208 tags of all samples), 10,640 tags for each sample were finally generated. The ultimate total dataset retained 166 OTUs at 99% similarity level. For taxonomically classified OTUs of UCYN-A sublineages, representative sequences and alignment sequences were aligned with ClustalW in MEGA v7.0. A maximum likelihood (ML) tree based on the Tamura-Nei model was then constructed. The node support of the ML tree was determined with 1,000 bootstrap replicates, and bootstrap values greater than 50% were shown.

### Determining UCYN-A Abundances Using Quantitative Polymerase Chain Reaction

The *nifH* genes for UCYN-A1 (Church et al., 2005) and UCYN-A2 (Thompson et al., 2014) were quantified using qPCR assays with TaqMan^®^ chemistry. It is notable that the UCYN-A2 assay is now known to also amplify the UCYN-A2/A3/A4 sublineages due to cross-reactivity between assays (Farnelid et al., 2016). Primer and probe sequences for the UCYN-A1 *nifH* gene assay are as follows: Forward primer (5′–3′) AGCTATAACAACGTTTTATGCGTTGA; Reverse primer (5′–3′) ACCACGACCAGCACATCCA; Probe, 5′-FAM-T CTGGTGGTCCTGAGCCTGGA-TAMRA-3′. For the UCYN-A2/A3/A4 *nifH* assay, the same reverse primer is used, and the forward primer and probe sequences are as follows: Forward primer (5′–3′) GGTTACAACAACGTTTTATGTGTTGA; Probe, 5′-FAM-TCTGGTGGTCCTGAGCCCGGA-TAMRA-3′. Preparation of standards, reaction conditions, and thermocycling parameters are described in Goebel et al. (2010), but it is important that primer probe specificity is ensured by setting different annealing temperatures of 60°C (UCYN-A1) and 64°C (UCYN-A2/A3/A4). The *r*^2^ values of the standard curves ranged from 0.98 to 1.00, and PCR efficiency ranged from 96 to 110%. All samples, standards, and inhibition tests were 10-μl qPCR reactions in duplicate or triplicate, and non-target templates were also tested under the same conditions. Based on the volumes of seawater filtered, as well as the DNA extract and DNA template volumes in the qPCR reactions, the limit of detection (LOD) and limit of quantitation (LOQ) in this study ranged between 25 and 154 *nifH* copies L^–1^, and between 200 and 1231 *nifH* copies L^–1^, respectively (Goebel et al., 2010). Samples with abundances greater than the LOD but less than the LOQ were labeled as “detected not quantified” (DNQ).

### Statistical Analysis

All statistical analyses were performed in R (v3.6.1^1^), using the “vegan” (Oksanen et al., 2015), “stats” (Field et al., 2012), “picante” (Kembel et al., 2010), “NST” (Ning et al., 2019), “hmisc” (Harrell and Dupont, 2013), “igraph” (Csardi and Nepusz, 2006), “gbmplus” (De’ath, 2007), and “ggplot2” (Wickham, 2016) packages.

### Spatial Variation Based on UCYN-A Community Diversity Indices

Alpha-diversity indices including richness, Chao1 richness, Ace richness, Pielou’s diversity, Shannon diversity, and the Simpson diversity index, were calculated at the OTU level using the “vegan” package (Oksanen et al., 2015). Kruskal–Wallis tests (Field et al., 2012) were used to compare the spatial differences of alpha-diversity indices. To interpret the spatial distribution patterns of UCYN-A communities and the differences among samples, non-metric multidimensional scaling (NMDS) was performed to represent UCYN-A community characters according to Bray–Curtis dissimilarities of the sequencing data. Differences in UCYN-A communities between region-based groups were investigated using permutational multivariate analysis of variance (PERMANOVA, permutations = 9999).

### The Assembly Processes of UCYN-A Communities

To evaluate the relative importance of deterministic or stochastic process on UCYN-A communities, we performed various statistical methods, including the neutral community model (NCM) (Sloan et al., 2006), and two types of null models from Stegen et al. (2013) and Ning et al. (2019) using the *nifH* amplicon dataset.

The NCM was applied to assess the contribution of stochastic processes to community assembly. This model predicts OTUs occurrence frequency in local communities (i.e., the UCYN-A community at each station) and their relative abundance across the wider metacommunities (i.e., the UCYN-A communities of total amplicon dataset) by the parameter *m*, which assesses the probability of a random loss being replaced by dispersal (Sloan et al., 2006; Burns et al., 2016). In this model, abundant taxa tend to be dispersed randomly and widespread across metacommunity, while rare taxa tend to disappear from local communities as a result of ecological drift. The parameter *R*^2^ reflects the overall fit of UCYN-A OTUs to NCM. The 95% confidence interval around the NCM prediction was used to identify which UCYN-A taxa were well predicted by this model (Burns et al., 2016).

In addition, a null modeling approach was applied using the framework as Stegen et al. (2013) described. Phylogenetic depths of environmental responses of the OTUs in the UCYN-A community were first estimated using Blomberg’s *K via* the “multiPhylosignal” function. Higher *K* values indicate a better correlation between species and phylogeny as expected by a Brownian motion-based metric of the strength of phylogenetic signal (Blomberg et al., 2003). *P* values for each *K* value were also calculated to test for significant phylogenetic signal, according to the variance of the phylogenetically independent comparisons deviated from a null model that randomly reshuffles trait values, based on 999 simulations. Subsequently, the pairwise phylogenetic turnover (βMNTD, quantifying the phylogenetic distance for each OTU in the community) was calculated using the “comdistnt” function in the “picante” package (Kembel et al., 2010). β-nearest taxon index (βNTI) is introduced to quantify the degree to which βMNTD deviates from the turnover expected, using a separate null model repeating the randomization 999 times (Stegen et al., 2012). βNTI measures the phylogenetic turnover given stochastic and deterministic ecological processes (Stegen et al., 2012; Wang et al., 2013; Dini-Andreote et al., 2015). | βNTI| > 2 indicates the dominance of niche-based processes, whereas | βNTI| < 2 indicates the influence of neutral processes. For the deterministic processes, βNTI > + 2 indicates variable selection processes and βNTI < −2 indicates homogeneous selection processes. Bray–Curtis-based Raup–Crick (RC_Bray_) was further employed to quantify neutral processes when | βNTI| < 2 (Chase et al., 2011; Stegen et al., 2013). RC_Bray_ > + 0.95 indicates a dominant role for dispersal limitation, whereas RC_Bray_ < −0.95 indicates a dominant process of homogenizing dispersal. The fractions of | βNTI| < 2 and | RC_Bray_ | < 0.95 indicate “undominated” processes, including weak selection, weak dispersal, and diversification or drift processes (Zhou and Ning, 2017; Tripathi et al., 2018).

To further enhance our understanding of the assembly process in microbial communities, another null model was applied using the framework as Ning et al. (2019) reported. In this framework, deterministic factors can drive the communities to be more similar or dissimilar than the null expectation, resulting in two types of selection processes. An index, named the normalized stochasticity ratio (*NST*), was inferred to quantify the deterministic (<50%) and stochastic assembly (>50%) by setting 50% as the boundary point. Multiple null model algorithms and community similarity metrics were applied in the current study to obtain a more comprehensive assessment.

### The Drivers Mediating UCYN-A Assembly

To evaluate the importance of each environmental variable on the UCYN-A community, we performed the aggregated boosted tree (ABT) analysis (De’ath, 2007), the Mantel test, and the co-occurrence network (CON) analyses using the *nifH* amplicon dataset.

The ABT analysis was employed to quantify the effect of each environmental variable on the UCYN-A community composition using the “gbmplus” package with 500 trees for boosting (De’ath, 2007). The Mantel test was also used to clarify significant environmental traits influencing the UCYN-A community *via* the “vegan” package (Oksanen et al., 2015).

Subsequently, CON including the correlations of OTUs vs. OTUs and OTUs vs. environmental factors was constructed. A total of 99 OTUs (more than 10 sequences) and 12 detected variables were selected to calculate pairwise Spearman’s rank correlations (*r*) within the “hmisc” R package (Harrell and Dupont, 2013). The final network consisted of all robust (*r* > 0.5 or *r* < −0.5) and statistically significant (*p* < 0.05) correlations. To obtain the pure species coexistence relationship in the UCYN-A communities, subnetwork analyses of OTUs belonging to total UCYN-A community or each sublineage were performed separately using the induced_subgraph function in the “igraph” package (Csardi and Nepusz, 2006). The node-level topological features, such as betweenness centrality, closeness centrality, degree, and eigenvector centrality, were calculated for all nodes. Betweenness centrality indicates the potential influence of a particular node on the connections of other nodes; the closeness centrality suggests the average distance of a node to any other node; degree represents the number of connections for a particular node with all neighboring nodes; and the eigenvector centrality assigns relative scores to all nodes in the network according to the concept that compared with low-scoring nodes, the connection of nodes with high scores contributes more to the score of a node in question (Greenblum et al., 2012; Berry and Widder, 2014). A core position of a node in the network is measured by high values of these topological features, while a peripheral position is reflected by low values of topological features (Ma et al., 2016; Jiao et al., 2017). Environmental variables that significantly correlated with OTUs could be seen as important niche-based factors. Networks were visualized based on Cytoscape 3.8.2.

## Results

### Biogeographic Diversity of UCYN-A From the Amplicon Dataset

**FIGURE 1:**
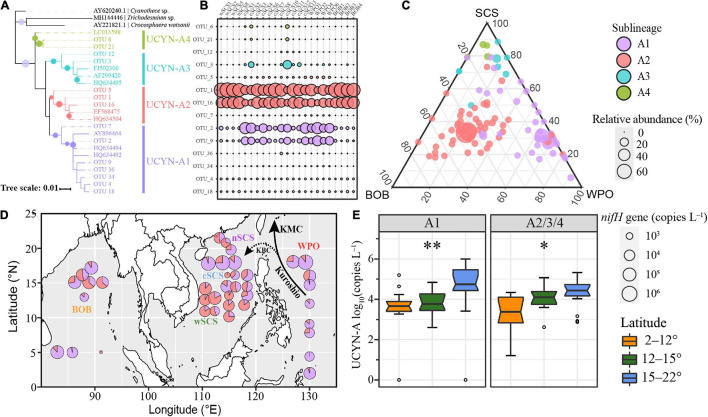
The biogeography and diversity of UCYN-A in the BOB, SCS, and WPO. **(A–C)** are based on the amplicon dataset. **(A)** Phylogenetic relationship of dominant OTUs belonging to UCYN-A1, UCYN-A2, UCYN-A3, and UCYN-A4, respectively. The bootstrap supporting values above 50 are indicated with solid circles. **(B)** The relative abundance of these OTUs across different stations. **(C)** The regional distribution of UCYN-A OTUs according to the ternary plot. The size of circles is proportional to the relative abundance of corresponding sublineages. **(D)** Abundance of UCYN-A1 and UCYN-A2/A3/A4 in the tropical seas based on the qPCR results. The size of circles is proportional to the log-transformed abundance of UCYN-A. **(E)** The latitudinal constraint of UCYN-A abundance. Significant differences were determined from the output of non-parametric Kruskal–Wallis rank-sum tests (^∗^*p* < 0.05; ^∗∗^*p* < 0.01).

An amplicon dataset with 1,395,208 high-quality sequences was obtained. The flat rarefaction curve indicates that the sequencing depths were sufficient to represent the diversity of local UCYN-A communities (Supplementary Figure 1). OTUs with more than 10 sequences were selected to represent UCYN-A communities. Thirty-six OTUs representing 23.81% of all sequences were clustered as UCYN-A1, and 51 OTUs contributing 72.30% of the dataset were affiliated to UCYN-A2. In fact, several OTUs together accounted for 96.6% of all sequences recovered, clustering closely within UCYN-A1/A2/A3/A4 (Figure 1A).

The distribution patterns of distinct sublineages were obviously different in the tropical seas. UCYN-A1/A2 dominated the dataset, while high relative abundances of UCYN-A3/A4 were only observed at several stations, such as station cSCS3 (Figure 1B). OTU 1 and OTU 16 dominated UCYN-A2, accounting for 42.0 and 28.2% of the UCYN-A *nifH* amplicon dataset, respectively. The OTUs of UCYN-A2 had a high proportion of relative abundance (average 72.3%), possessing a central position in the ternary plot (Figure 1C). Furthermore, UCYN-A2 was particularly abundant (relative abundance > 95%) at some stations in the BOB and wSCS.

The phylogenetic tree contained seven OTUs (OTU 2, OTU 4, etc.) closely related with UCYN-A1 reference sequences (bootstrap value 67%). OTU 2 and OTU 9 dominated UCYN-A1, accounting for 13.2 and 8.1% of all sequences, respectively. UCYN-A3 was widely distributed, and its relative abundance was 22% in a coastal station near Hainan Island (cSCS10) (Supplementary Figure 2). Meanwhile, at this station, the relative abundance of UCYN-A4 (6.7%) was about 20 times higher than that in other regions.

**FIGURE 2:**
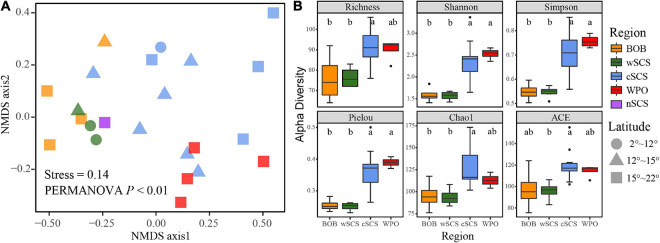
Spatial heterogeneity of UCYN-A communities in three contrasting seas. **(A)** Non-metric multidimensional scaling (NMDS) analysis revealed the spatial variation of UCYN-A. Significant difference was determined by PERMANOVA with 9,999 permutations. **(B)** The alpha diversity indices across different regions. Boxplots that do not share a letter are significantly different (*p* < 0.05; non-parametric Wilcoxon rank-sum test).

Distinct biogeographic patterns emerged when the distribution pattern of NMDS and diversity indices were divided by regions (Figure 2). PERMANOVA confirmed the statistical significance of the region-based group (permutations = 9,999, *p* < 0.05). The difference of beta-diversity implied the spatial differentiation of UCYN-A. Alpha-diversity indices in the WPO and cSCS were significantly higher than those in the wSCS and BOB (Wilcoxon rank-sum test, *p* < 0.01). Moreover, high-latitude areas presented higher alpha-diversity indices than did low-latitude areas (Supplementary Figure 3).

### Biogeographic Diversity of UCYN-A From qPCR

Significant variation in UCYN-A1/A2 abundances was observed among distinct latitudes rather than different regions (Figures 1D,E and Supplementary Figure 4). UCYN-A1 had higher mean qPCR-based abundances than did UCYN-A2/A3/A4 (8.4 × 10^4^ *nifH* copies L^–1^, including non-detects), with two samples below the LOD and one sample below the LOQ. The highest UCYN-A1 abundance, at 9.98 × 10^6^ *nifH* copies L^–1^, occurred at station WPO-6. UCYN-A2/A3/A4 mean abundances were lower (2.5 × 10^4^ *nifH* copies L^–1^, including non-detects) than those of UCYN-A1, with only one sample below the LOD. The highest UCYN-A2/A3/A4 abundances, at 2.1 × 10^6^ *nifH* copies L^–1^, occurred at station cSCS-2. Generally, UCYN-A abundances peaked in several stations close to 18°N, such as station WPO-6, and were lower toward the lower latitudes.

The ratio of UCYN-A1/A2 based on abundances and relative abundance shared a similar trend (Pearson’s *r* = 0.82) (Figure 1 and Supplementary Figure 2). Both methods indicated that the proportion of UCYN-A1 tended to be higher near the Kuroshio Current and lower after dispersal to remote areas or mixing with local waters. This biogeographic pattern suggested a potential stochastic effect on UCYN-A distribution and diversity.

### Quantifying Influences of Ecological Processes in Shaping Community Structure

**FIGURE 3:**
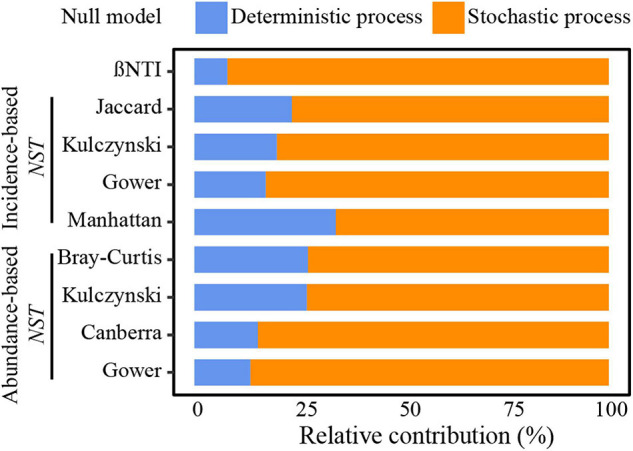
The relative effect of deterministic and stochastic processes governing the UCYN-A community assembly. The contribution was evaluated by various null models with different algorithms and community similarity indices.

A large proportion of | βNTI| values were lower than 2 and all *NST* values from different algorithms and community similarity metrics were >50%, indicating that stochastic processes mainly governed community dynamics (Figure 3). The relative contribution of stochasticity was 79–91% for UCYN-A1 and 80–95% for UCYN-A2 (Supplementary Figure 5 and Supplementary Table 4). Additionally, there was a high proportion of homogenizing dispersal (49.8%) and weak-ecological processes (40.7%) in the overall community. Variable selection processes (7.9%) seemed to be more important than dispersal limitation (1.6%) in UCYN-A communities, and a relatively high proportion of variable selection processes was estimated in UCYN-A2 (6.7%) compared with UCYN-A1 (5.5%). In addition, a relatively small proportion of homogeneous selection processes was calculated in UCYN-A2 (non-detected) than in UCYN-A1 (3.2%).

### The Fit of Neutral Processes

**FIGURE 4:**
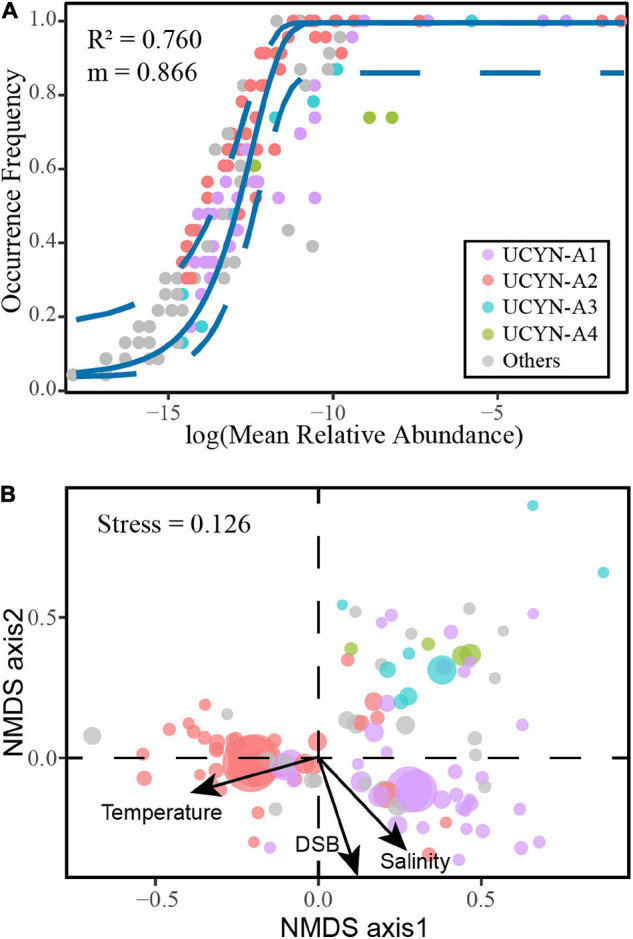
The neutral-based and niche-based processes of UCYN-A communities assessed by multiple models. **(A)** The fit of neutral community model (NCM) to UCYN-A communities. **(B)** Non-metric multidimensional scaling (NMDS) analysis.

With 76.0% of community variance explained, the NCM successfully estimated a large proportion of the relationship between OTUs occurrence frequency and their variation in relative abundance (Figure 4A), indicating the importance of stochastic processes in shaping the community assembly. Moreover, neutral and non-neutral partitions are genetically and ecologically distinct (Supplementary Table 5). Interestingly, two OTUs below the NCM confidence interval accounted for 93.4% of UCYN-A4 relative abundance. The strong deviation of UCYN-A4 indicated that the assembly mechanism of UCYN-A4 was significantly different from other sublineages. Furthermore, the *m* value was estimated to be 0.866, suggesting strong species dispersal processes.

### The Niche-Based Drivers Shaping the Biogeography of UCYN-A1 and UCYN-A2

**FIGURE 5:**
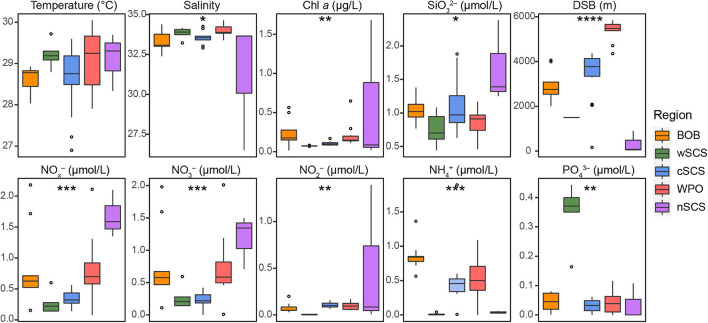
Spatial variation of environmental parameters in three tropical seas. Environmental variables include temperature, salinity, chlorophyll *a* (Chl *a*), silicate (SiO_3_^2–^), the distance from sea surface to bottom (DSB), ammonium (NH^4+^), nitrate (NO_3_^–^), nitrite (NO_2_^–^), nitrate + nitrite (NO_*x*_^–^), and phosphate (PO_4_^3–^). Significant differences were investigated using non-parametric Kruskal–Wallis rank-sum tests (*p < 0.05; **p < 0.01; ***p < 0.001; ****p < 0.0001).

Some environmental traits (e.g., nutrients) among different regions were significant in the current study (Figure 5). Although niche-based processes played a minor role in community assembly in this study, several environmental factors significantly altered the community structure. Based on the species scores in NMDS, closely related UCYN-A taxa had more similar niche preferences (Figure 4B). We further evaluated trait conservatism in response to environmental changes (Supplementary Table 6). The UCYN-A community exhibited a strong and significant phylogenetic signal for temperature (*K* = 0.567; *p* < 0.001). This indicates that within a UCYN-A community, closely related taxa had more similar responses to temperature.

**FIGURE 6:**
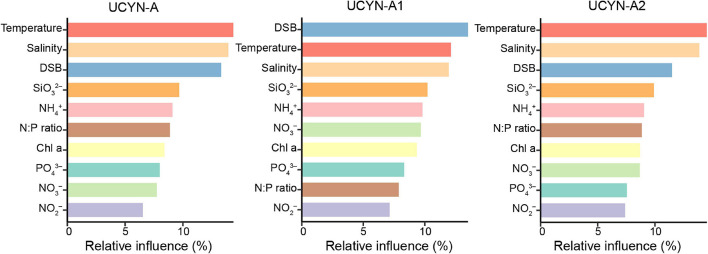
Aggregated boosted tree (ABT) analysis shows the relative effect of each environmental factor on UCYN-A community composition. Environmental variables include temperature, salinity, the distance from sea surface to bottom (DSB), silicate (SiO_3_^2–^), ammonium (NH^4+^), chlorophyll *a* (Chl *a*), nitrate (NO_3_^–^), nitrite (NO_2_^–^), phosphate (PO_4_^3–^), and N:P ratio (N/P).

**FIGURE 7:**
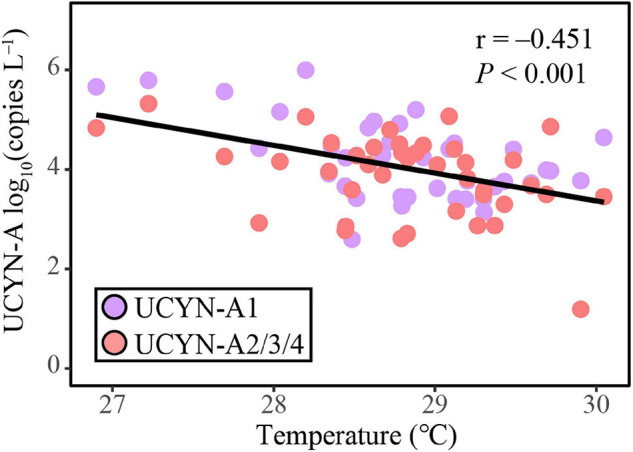
The correlation between UCYN-A abundance and sea surface temperature, estimated by linear least-squares regression.

The ABT model was further applied to interpret the relative importance of environmental parameters on the composition of UCYN-A communities. The results were consistent with the NMDS ordination and the Mantel test, where temperature (Mantel test, *R* = 0.486, *p* < 0.001), coastal/oceanic habitats associated with salinity (Mantel test, *R* = 0.211, *p* < 0.05), and DSB (Mantel test, *R* = 0.330, *p* < 0.01) were identified as primary factors directly or indirectly correlated with the changes in community composition (Figures 4, 6 and Supplementary Table 7). ABT verified that sea surface temperature was the most important factor influencing the diversity of the UCYN-A community. ABT also clearly showed that salinity and DSB exerted significant influence on the UCYN-A community. Meanwhile, UCYN-A abundances decreased as temperature (26.9–30.0°C) increased (*r* = −0.451, *p* < 0.001) in the tropical seas (Figure 7). Additionally, the ratio of UCYN-A1/A2 abundance was significantly positively correlated with salinity (*r* = 0.387, *p* < 0.05) and DSB (*r* = 0.335, *p* < 0.05). Interestingly, the most important drivers varied with different sublineages, implying that UCYN-A1 and UCYN-A2 are distinct ecotypes.

## Discussion

### Distinct Assembly Mechanisms Shaping the Spatial Heterogeneity of UCYN-A

Little is known about the assembly mechanisms underpinning the diversity and biogeography of UCYN-A communities. The NMDS and PERMANOVA analyses showed that samples from the same region (*p* < 0.05) clustered more closely than those from the same latitude (*P* = 0.14), suggesting the significant genetic diversity of UCYN-A from different tropical seas.

This distinct distribution pattern can be the result of stochastic community assembly processes. The null models were employed to quantify the ecological processes, and the findings that stochastic processes dominated UCYN-A community assembly were consistent with the results of a soil study conducted in bacterial communities across the North China Plain (Shi et al., 2018), as well as a study focused on methanotrophs communities in the Qinghai-Tibetan Plateau (Zhang et al., 2019). Due to geographical isolation and/or large-scale geographical distance, dispersal limitation processes may influence the generation and maintenance of microbial community diversity (Zhou and Ning, 2017) across different seas. Despite the low contribution of dispersal limitation in the current study, the significantly increased βNTI values with increasing geographical distance (Supplementary Figure 6) suggest that determinism may overwhelm stochasticity across a broad spatial scale of more than 5,000 km, which is consistent with a previous assumption that spatial scale influences the importance of niche and neutral processes (Chase, 2014). This large scale is significantly greater than the turning point of determinism/stochasticity reported previously in plateau (130 km) and plain (900 km) environments (Shi et al., 2018). This difference may come from the more similar environmental pressures in the waters compared with those in the highly variable soil habitats across the same spatial scale, as well as the distinct dispersal rates under the combined effect of a different dissemination medium (waters or soils) and microbial carrier (bacteria or symbionts). Meanwhile, the transportation of ocean currents and water masses can deliver microbes to distant regions and cause high levels of microbial dispersal, alleviating the dispersal limitation along the current path. Both of the qPCR-derived UCYN-A *nifH* gene abundances and *nifH* amplicon assays indicate a declining trend of UCYN-A1 proportion along the Kuroshio Current intrusion, suggesting a significant role of ocean currents on the spatial distribution pattern of microbes in these areas (Figure 1; Shiozaki et al., 2013, 2018). Indeed, it has been suggested that diazotrophs can remain active after being transported over great distances with ocean currents (Shiozaki et al., 2013). In addition, wind direction and speed have been identified as important environmental factors influencing the composition and abundance of UCYN-A sublineages in the Western Tropical South Pacific (Henke et al., 2018). Although it is challenging to evaluate the influences of stochasticity, it is of vital importance to incorporate physical parameters (e.g., the direction, velocity, and volume of ocean currents) into marine ecological frameworks in the future.

The NCM is an extensively applied method for inferring stochastic processes acting on community assembly (Burns et al., 2016; Zhou and Ning, 2017; Chen W. et al., 2019). This model is used to depict the importance of neutral processes, which are difficult to observe directly but have significant influences on microbial communities (Tong et al., 2019). A major part of the community variation is estimated by the NCM, suggesting that stochastic processes (e.g., random births, deaths, and immigrations) are critical in shaping the community assembly. Several important observations based on the NCM also revealed similar results to our finding. For instance, Chen W. et al. (2019) investigated the microeukaryotic communities during wet and dry seasons in the Tingjiang River, and found that microeukaryotic communities were strongly driven by stochastic processes (*R*^2^ = 0.90 explained by the NCM). Moreover, Roguet et al. (2015) revealed that bacterial community structure was mainly driven by stochastic processes (*R*^2^ = 0.76 explained by NCM) in 49 lakes in Paris, France (Roguet et al., 2015). Accordingly, the stochastic processes are powerful enough to generate significant community diversity among tropical seas.

Our study highlights the importance of stochastic processes on microbial community assembly in marine ecosystems. However, both null model and neutral community model methods have an intrinsic problem, that an obtained result fitting a null/neutral model cannot reject the importance of deterministic processes, since a niche model could present a result resembling that of null/neutral models (Zhou and Ning, 2017). Therefore, habitat filtering, to some extent, structures the microbial community. The difference in some environmental traits (e.g., Chl *a* and nutrients, bottom-up control) among different regions was significant in the present study (Figure 5). For instance, the wSCS region was affected by upwelling (Voss et al., 2006). The high concentrations of phosphate and low N/P ratios here may both directly and indirectly influence the diazotrophic community composition (Zhang et al., 2015). We also detected strong correlations between the UCYN-A community structure and the position of coastal/oceanic habitats across different stations. In addition, biotic interactions can shape a distinct community structure. For example, the C-N exchange of UCYN-A/haptophytes may enhance their ability to survive in a variety of habitats. Recently, a co-occurrence relationship between UCYN-A1 and UCYN-A3 has been reported (Turk-Kubo et al., 2017). Our NMDS and CCN results further confirmed this co-existence, as UCYN-A1 and UCYN-A3 clustered closely with significantly positive correlations among all corresponding OTUs (Figure 4B; Supplementary Figure 7; and Supplementary Table 8). All correlations between UCYN-A2 and UCYN-A4 were also positive, which is consistent with the potential co-occurring relationship between UCYN-A2 and UCYN-A4 as previously reported (Turk-Kubo et al., 2017). In fact, UCYN-A3 and UCYN-A4 are rare taxa compared with UCYN-A1 and UCYN-A2, thus likely possessing narrower niches in the global ocean. However, the ecological roles of these rare species should not be neglected because they can provide cover for the selection from a “seed bank” (Caron and Countway, 2009; Konopka et al., 2015). Moreover, grazing (Scavotto et al., 2015) and viral infection (Wilhelm and Suttle, 1999) (top-down control) may influence the UCYN-A symbionts. Therefore, the combination of different abiotic and biotic drivers or stochasticity can shape the distinct community diversity and biogeography of UCYN-A in the tropical seas.

### The Drivers Mediating UCYN-A Assembly

High UCYN-A abundances were detected in the tropical seas, yet the abundances were higher near the subtropical area. This result agrees with a previous study in the WPO and SCS that higher UCYN-A abundances mainly occurred between 18 and 20°N (Chen M. et al., 2019). This slight but statistically significant latitudinal constraint on UCYN-A abundances suggest that although niche-based processes are relatively weak in the tropical seas, their effects are also important in driving the biogeography of UCYN-A communities.

**FIGURE 8:**
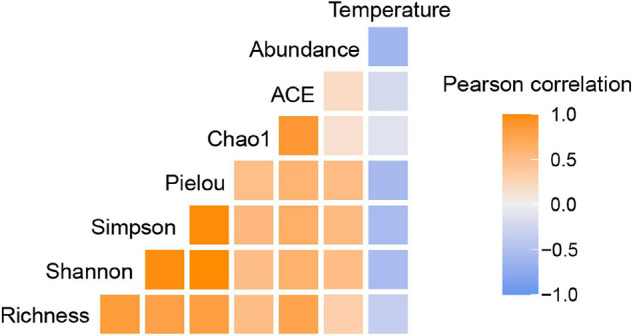
The correlation between temperature and abundance/diversity of UCYN-A, revealed by Pearson correlation using the amplicon dataset and qPCR results. Six alpha-diversity indices were used to represent microbial diversity.

Temperature may be responsible for the latitudinal constraint on the abundance of UCYN-A as discussed above. As a fundamental environmental variable, temperature can determine the composition and function of microbial communities. It has been suggested that the global biogeographical distribution of marine diazotrophic cyanobacteria reflects the physiologically preferential temperature range at which they can perform nitrogen fixation (Brauer et al., 2013). However, it has not been clear how temperature mediates diazotrophic community assembly processes across the global ocean. Here, we reported an interesting taxa, UCYN-A, the temperature optima of which was previously inferred to be near 24°C (Moisander et al., 2010), to partly address this mystery. A phylogenetic signal in the ecological niches of OTUs was detected, suggesting that more closely related UCYN-A taxa have more similar niche preferences related to temperature (Losos, 2008). Moreover, the ABT model, NMDS ordination and Mantel test demonstrated that temperature was the most important environmental factor in driving the UCYN-A community variation observed in the current study. Correlation analysis also showed that the UCYN-A abundances and diversity indices were negatively correlated with temperature (26.9–30.0°C) (Figure 8), which is consistent with previous studies (Moisander et al., 2010; Bonnet et al., 2015; Turk-Kubo et al., 2015).

**FIGURE 9:**
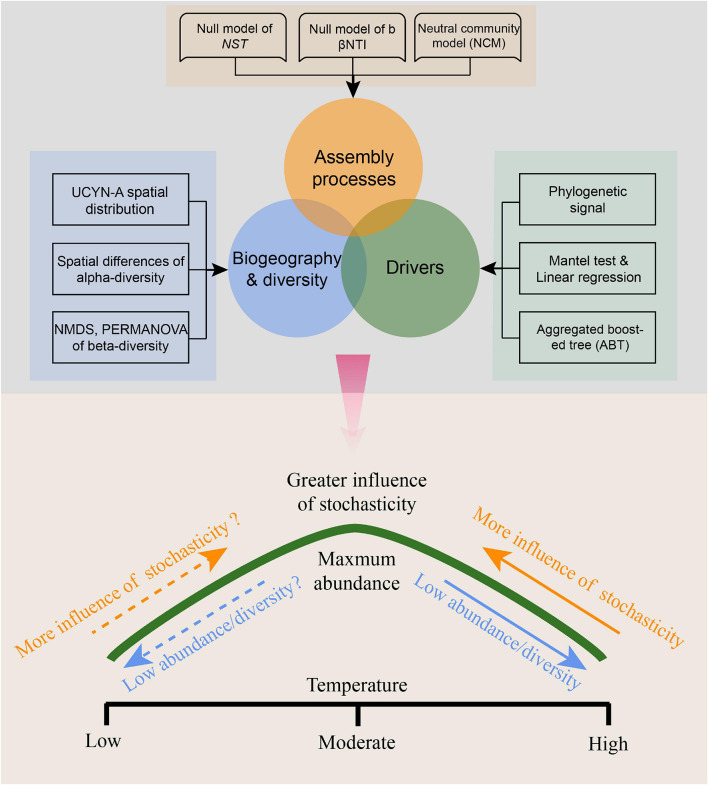
Conceptual model showing the variation of stochasticity and abundance/diversity of UCYN-A communities with changing temperature. The dotted line means this part of the model has not been confirmed and requires further research.

Null models using various community similarity algorithms showed similar results of inferred ecological processes (Table 1), indicating that temperature mediates the degree to which deterministic vs. stochastic processes shape UCYN-A community assembly. Our results are conceptually consistent with the reported findings of Tripathi et al. (2018) on bacteria and some studies on macro-organisms (Chase, 2007; Helmus et al., 2010; Myers and Harms, 2011). These results, coupled with previous paradigms (Tripathi et al., 2018), lead us to propose a conceptual model (Figure 9), which depicts how stochastic processes and abundance/diversity of UCYN-A communities change with sea surface temperature in the ocean. From the spatial perspective, a near-optimal temperature will lead to more stochastic assemblies and higher abundance/diversity in moderate latitude areas, and a shift in temperature toward relatively extreme conditions at low latitude will result in more deterministic assemblies and less abundance/diversity. While from the temporal perspective, extreme temperature in warmer seasons will lead to more deterministic assemblies and less abundance/diversity, and progressive shifts in temperature toward optima conditions will generate weaker selection, more stochasticity and higher abundance/diversity. However, although we suggest that the importance of neutral vs. niche-based assembly of UCYN-A communities is significantly influenced by temperature, the generality of this framework is largely limited by the narrow range of temperatures in the tropical seas and the uncertainty of temperature optima; there have been many detections of abundant UCYN-A in cold waters (Shiozaki et al., 2017, 2018; Harding et al., 2018). Therefore, it is necessary to test this conceptual model in different ecosystems spanning a large temperature range (e.g., including ecosystems in both high- and low-latitude regions).

**TABLE 1:** Stochasticity in UCYN-A community assembly estimated by different indices based on various similarity metrics.

	**Similarity metrics**	**High**	**Moderate**
Incidence-based	Jaccard	**0.608 ± 0.052**	**0.798 ± 0.038**
	Kulczynski	**0.622 ± 0.062**	**0.819 ± 0.04**
	Gower	**0.677 ± 0.054**	**0.846 ± 0.037**
	Manhattan	**0.482 ± 0.05**	**0.694 ± 0.051**
Abundance-based	Bray-Curtis	0.5650.154	0.6750.084
	Kulczynski	0.5630.159	0.6750.084
	Canberra	0.7690.062	0.8430.039
	Gower	0.6940.077	0.7930.082

*The distribution of *NST* at high temperatures and near-optimal temperatures was obtained from 999 permutations. The significance of *NST* differences between two groups was tested for using permutational ANOVA test with 999 permutations. Bold values indicate significant differences (*p* < 0.01).*

Ecologists and biogeographers might also focus on other factors affecting UCYN-A communities. There is no doubt that distinct habitats exert an effect on the processes of microbial community assembly. Mills et al. (2020) recently reported an unusual mechanism that UCYN-A/prymnesiophyte symbiosis relies on N_2_ fixation, meets little of its N demands *via* ammonium uptake, and does not assimilate nitrate even in N-rich environments. The implication is that N_2_ fixation of UCYN-A is important in global coastal and oceanic habitats. This unique N-utilization strategy also partly explained the high N_2_ fixation rates and UCYN-A abundance in the coastal region where nitrate concentrations were extremely high as Tang et al. (2020) reported. Moreover, the cross-feeding between UCYN-A and their hosts (Thompson et al., 2012; Zehr, 2015; Martinez-Perez et al., 2016) allows them to survive in a variety of habitats even under nutrient limitation and competitive conditions, so that the distribution of UCYN-A is more influenced by neutral processes.

Interestingly, in the current study, the ratio of UCYN-A1/A2 abundance (and relative abundance) positively correlated with salinity and DSB, suggesting the preference of UCYN-A2 (UCYN-A1) in coastal (oceanic) habitats. The UCYN-A2/*B. bigelowii* association (4–20 μm) is known to be larger than the UCYN-A1 association (1–3 μm) (Thompson et al., 2012, 2014; Cabello et al., 2016; Farnelid et al., 2016; Zehr et al., 2016; Cornejo-Castillo et al., 2019). These size differences are consistent with the theory that larger phytoplankton with higher abundances tend to be found in eutrophic conditions (Irwin et al., 2006). However, our findings of higher stochasticity for UCYN-A2 compared with UCYN-A1 (Supplementary Table 4) contradict a recent study that small microorganisms are relatively more influenced by stochastic processes (Luan et al., 2020). This contradiction may arise from the fact that the body size of UCYN-A symbionts is not a proper driver governing UCYN-A community assembly. Apart from temperature, other important deterministic parameters, such as light, iron availability, biotic grazing, and viral infection, have been shown to be of vital importance in the growth and metabolism of microbes (Wilhelm and Suttle, 1999; Ribalet et al., 2015; Scavotto et al., 2015; Bonnain et al., 2016). Determining how limiting effects of these factors definitively drive community variability cannot be discussed elaborately in the present study, yet quantifying the contribution of these factors using mathematical models has profound implications for further investigations.

## Conclusion

Our study provides the first example of research revealing both stochastic processes and deterministic processes driving the biogeography and diversity of marine UCYN-A communities. Dispersal limitation can cause distinct microbial biogeography, influencing the generation and maintenance of microbial community diversity. Meanwhile, the transportation of ocean currents and water masses may bring the high possibility of microbial immigration and promote the cross-regional distribution of microbial communities in the ocean. These stochastic processes hint at the urgency of incorporating parameters of physical processes into marine ecological frameworks in the future, and clarify the balance between microbial dispersal and dispersal limitations mediating microbial biogeography. On the other hand, we cannot reject the vital roles of deterministic processes given that a niche model could show a pattern similar to that of null/neutral models (Zhou and Ning, 2017). The ABT model, NMDS ordination, and Mantel test concurrently demonstrated that temperature and coastal/oceanic habitats associated with salinity and DSB were important environmental factors in shaping UCYN-A community assembly. We further propose a conceptual framework that temperature mediates species abundance/diversity and the degree of stochasticity/determinism. Although we suggest that our framework provides novel insights, it is important to recognize its limitations including that the framework was concluded based on the narrow temperature range of the tropical seas. It is promising to disentangle the relative importance of stochastic processes and deterministic processes and further propose a broader framework by integrating the global data including tropical, temperate, and polar environments into ecological models.

## Data Availability Statement

The raw data of *nifH* gene sequences have been submitted to the NCBI Sequence Read Archive (SRA) under Bioproject PRJNA650247. The authors acknowledge that the data presented in this study must be deposited and made publicly available in an acceptable repository, prior to publication. Frontiers cannot accept a manuscript that does not adhere to our open data policies.

## Author Contributions

CW, JS, and LL conceived and designed the experiments. LL, CW, and CD carried out the sample collection, extracted DNA, and performed the experiments. LL and DH analyzed the data. LL wrote the manuscript. LL, JS, CW, DH, and YW contributed to the manuscript development and revisions. All authors read and approved the final manuscript.

## Conflict of Interest

The authors declare that the research was conducted in the absence of any commercial or financial relationships that could be construed as a potential conflict of interest.

## Publisher’s Note

All claims expressed in this article are solely those of the authors and do not necessarily represent those of their affiliated organizations, or those of the publisher, the editors and the reviewers. Any product that may be evaluated in this article, or claim that may be made by its manufacturer, is not guaranteed or endorsed by the publisher.

## Footnotes

1
http://www.r-project.org/
