# Supplementary material for: Integrating Stochastic and Deterministic Process in the Biogeography of N2-Fixing Cyanobacterium Candidatus Atelocyanobacterium Thalassa
Source: Front Microbiol. 2021 Oct 21;12:654646. doi: 10.3389/fmicb.2021.654646 (PMC8566894; doi:10.3389/fmicb.2021.654646)
Supplement: Supplementary file 1 [file Data_Sheet_1.doc]

*Supporting information for the article:*

**Integrating stochastic and deterministic process in the biogeography of N_2_-fixing cyanobacterium *Candidatus* Atelocyanobacterium thalassa (UCYN-A)**

**Running title:** Assembly processes and biogeography of UCYN-A

**This supporting information contains:**

- 16 Pages
- 7 Figures
- 8 Tables
- 3 references


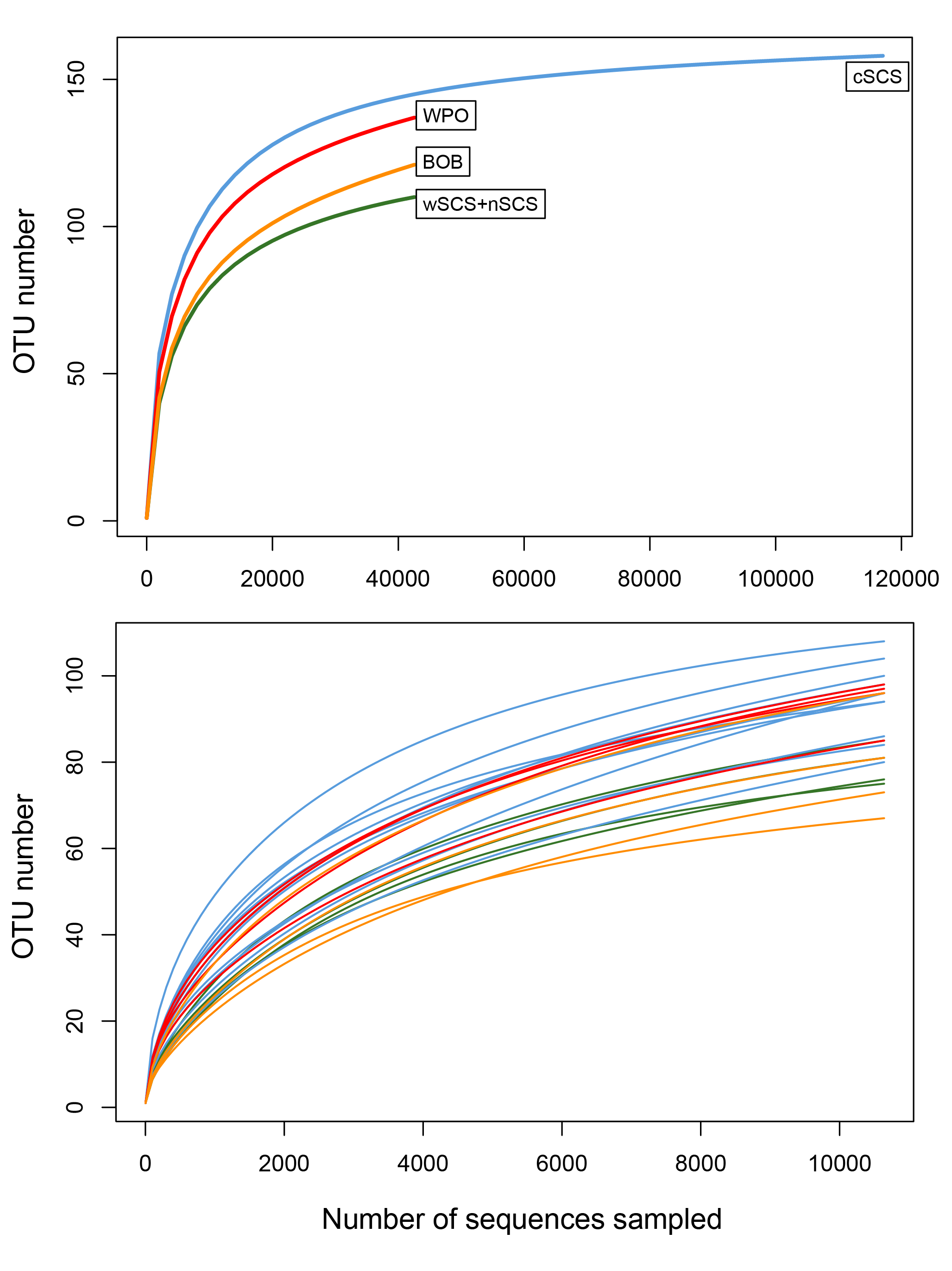


**Figure S1** Rarefaction curves of all samples from the different regions.


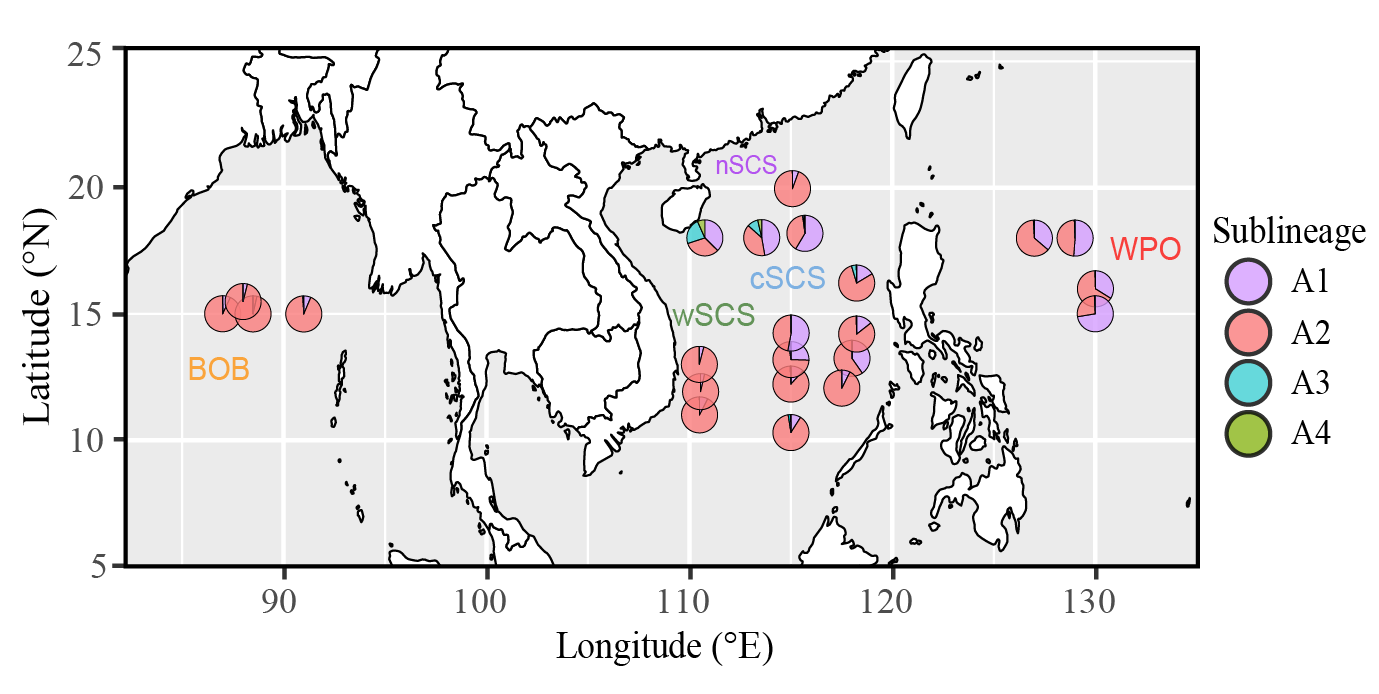


**Figure S2** Relative abundance of UCYN-A1, UCYN-A2, UCYN-A3, and UCYN-A4 in the BOB, SCS, and WPO.


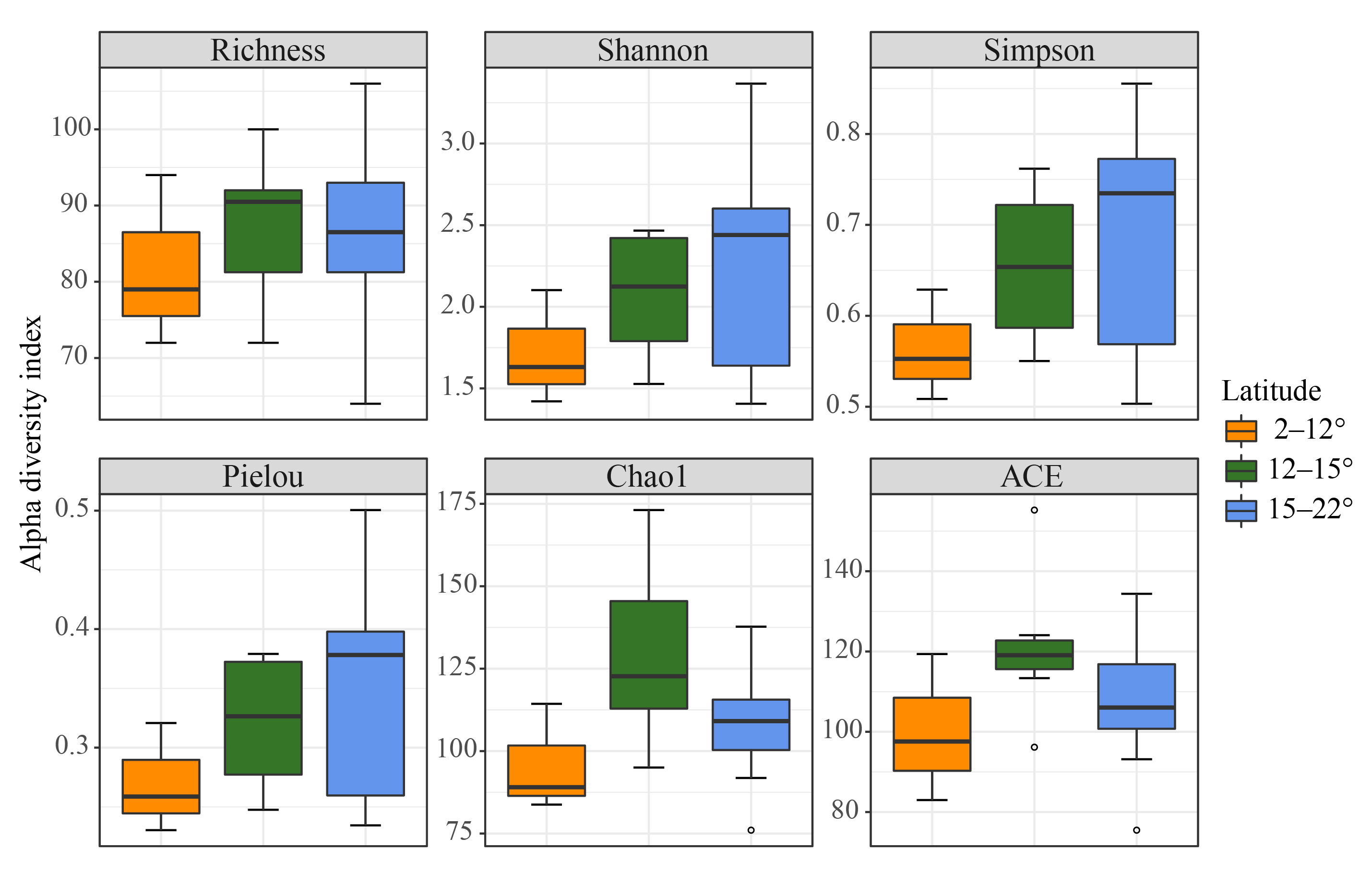


**Figure S3** Latitudinal constraint of alpha diversity indices of UCYN-A communities.


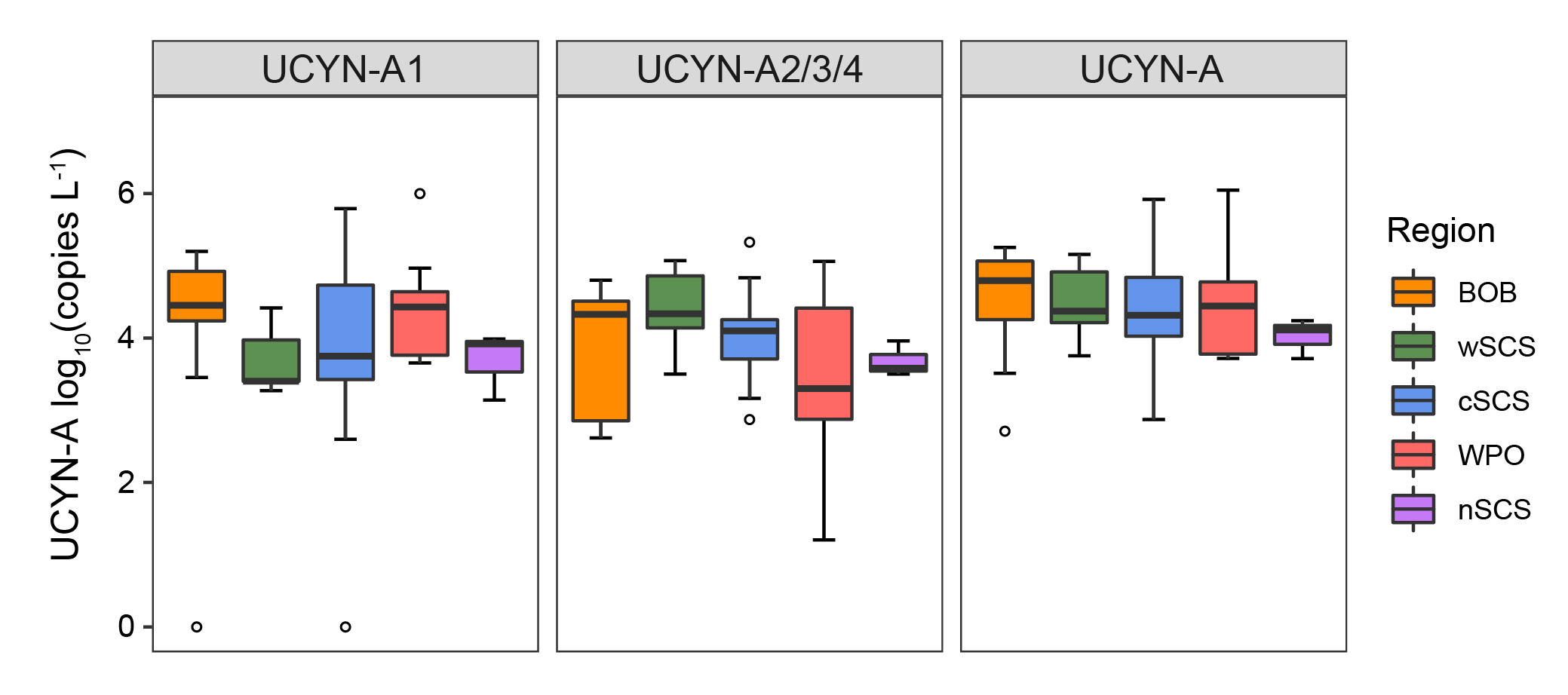


**Figure S4** Abundance of UCYN-A1, UCYN-A2/A3/A4, and UCYN-A in different regions.


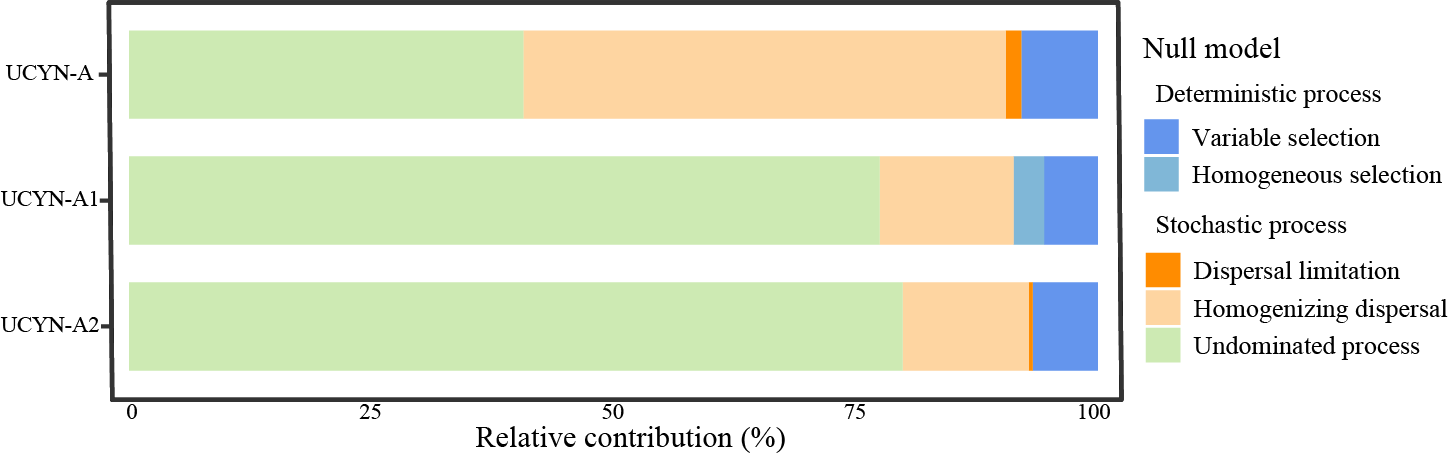


**Figure S5** The relative effect of various ecological processes governing the UCYN-A community. The percent of turnover in community assembly is controlled primarily by deterministic (homogeneous and variable selection) and stochastic processes (dispersal limitation, homogenizing dispersal, and the part that was not dominated by any single process).


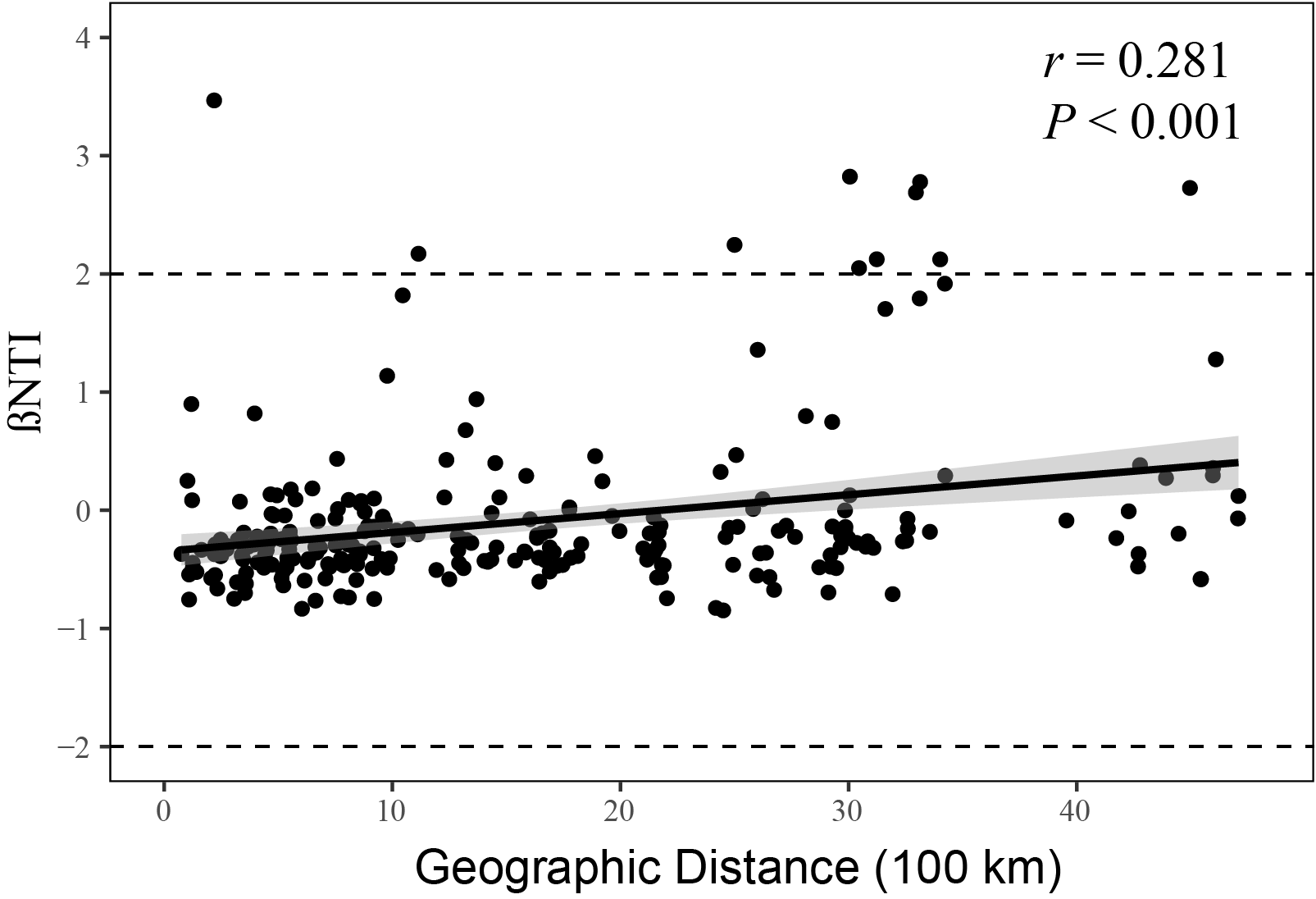


**Figure S6** βNTI values with geographical distance in three tropical seas.


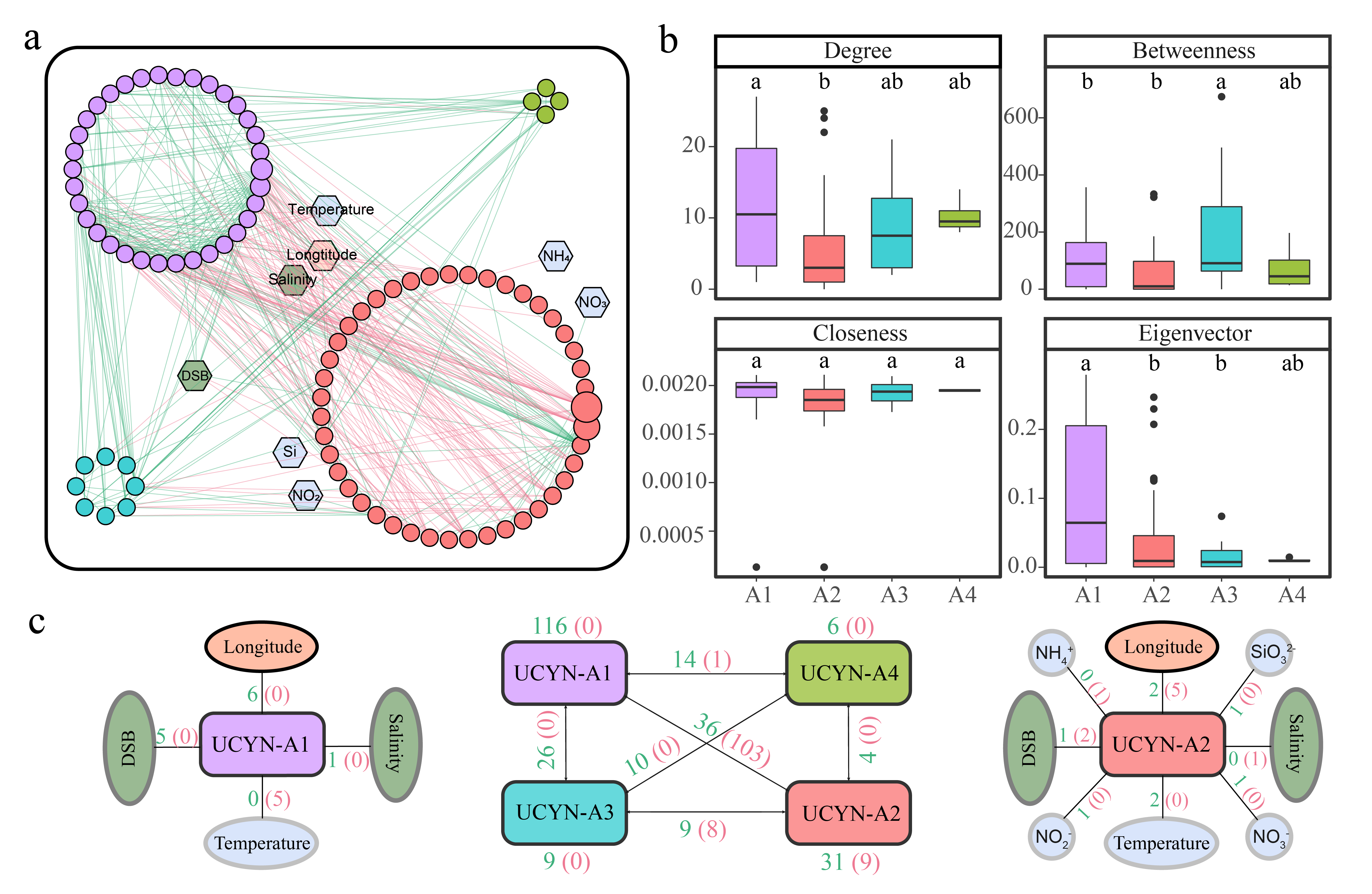


**Figure S7** Disentangling the niche-based processes by constructing the co-occurrence relationships. Co-occurrence network (CON) includes the correlations between OTUs, and between OTUs and environmental factors (a); Node-level topological features of different sublineages, specifically the degree, betweenness, closeness, and eigenvector centrality, from the subnetwork including only UCYN-A OTUs (b) and the network summary (c). The figure was colored based on UCYN-A sublineages and environmental or spatial factors. Each node in the network represents an OTU or external factor, and each edge indicates a correlation. The size of the OTUs-nodes is proportional to the relative abundance of corresponding OTUs. The red line indicates negative relationships and green line represents positive relationships. Numbers outside (inside) parentheses indicate the numbers of positive (negative) edges. Boxplots that do not share a letter are significantly different (*P* < 0.05; non-parametric Wilcoxon rank-sum test).

**Table S1 Basic information of PCR primers used in the first amplification and second amplification**

| Primers | Sequences |
| --- | --- |
| nifH3 | 5’-TTYTAYGGNAARGGNGG-3’ |
| nifH4 | 5’-ATRTTRTTNGCNGCRTA-3’ |
| univ_UCYN-A_F_CS1 | 5’-ACACTGACGACATGGTTCTACAAGTTTGCATTGTAAAGCACA-3’ |
| univ_UCYN-A_R_CS2 | 5’-TACGGTAGCAGAGACTTGGTCTTCCTTCAC GGATAGGCATAG-3’ |

**Table S2 Reaction system for nested PCR analysis.**

| Reaction mixtures | Round 1 | Round 2 |
| --- | --- | --- |
| 10 × PCR buffer | 10 μL | 10 μL |
| dNTPs | 400 mM | 400 mM |
| BSA | 0.5 μL | 0.5 μL |
| Forward primer (10 μM) | 0.5 μL (nifH3) | 0.5 μL (univ_UCYN-A_F_CS1) |
| Reverse primer (10 μM) | 0.5 μL (nifH4) | 0.5 μL (univ_UCYN-A_R_CS2) |
| KOD FX Neo polymerase | 0.2 μL | 0.2 μL |
| Template DNA | 1 μL | 1 μL |

**Table S3** The procedure of PCR analysis.

|  | First round | Second round |
| --- | --- | --- |
| Initial denaturation | 95℃, 5 min | 95℃, 5 min |
| Denaturation | 94℃, 1 min | 95℃, 30 s |
| Annealing | 52℃, 1 min | 55℃, 30 s |
| Extension | 72℃, 1 min | 72℃, 30 s |
| Cycles | 38 | 25 |
| Final extension | 72℃, 7 min | 72℃, 7 min |
| Storage temperature | 4℃ | 4℃ |

**Table S4** Stochasticity in UCYN-A community assembly estimated by different indices based on various similarity metrics. The distribution of *NST* was obtained from 999 permutations.

|  | Similarity metrics | UCYN-A1 | UCYN-A2 |
| --- | --- | --- | --- |
| Incidence-based | Jaccard | 0.790 ± 0.054 | 0.890 ± 0.024 |
|  | Kulczynski | 0.820 ± 0.060 | 0.929 ± 0.022 |
|  | Gower | 0.879 ± 0.033 | 0.934 ± 0.018 |
|  | Manhattan | 0.821 ± 0.042 | 0.836 ± 0.029 |
| Abundance-based | Bray-Curtis | 0.887 ± 0.054 | 0.948 ± 0.058 |
|  | Kulczynski | 0.891 ± 0.060 | 0.944 ± 0.063 |
|  | Canberra | 0.797 ± 0.051 | 0.915 ± 0.023 |
|  | Gower | 0.855 ± 0.038 | 0.804 ± 0.036 |

**Table S5** Stochastic processes varied with different UCYN-A sublineages

|  | Prediction | UCYN-A1 | UCYN-A2 | UCYN-A3 | UCYN-A4 |
| --- | --- | --- | --- | --- | --- |
| Richness | Above | 4 | 20 | 0 | 0 |
|  | Neutral | 27 | 30 | 7 | 2 |
|  | Below | 5 | 1 | 1 | 2 |
|  | Neutral proportion | 75% | 59% | 88% | 50% |
| Relative abundance | Above | 0.02% | 0.61% | 0.00% | 0.00% |
|  | Neutral | 23.50% | 71.65% | 1.97% | 0.04% |
|  | Below | 0.28% | 0.03% | 0.06% | 0.55% |
|  | Neutral proportion | 98.73% | 99.11% | 96.83% | 6.61% |

**Table S6** Phylogenetic signal showing the level of trait conservatisms for environmental responses of OTUs in UCYN-A1, UCYN-A2 and total UCYN-A communities using Blomberg’s *K*. Environmental factors include temperature, salinity, silicate (SiO_3_^2–^), ammonium (NH^4+^), chlorophyll *a* (Chl *a*), nitrate (NO_3_^–^), nitrite (NO_2_^–^), and phosphate (PO_4_^3–^).

|  | UCYN-A | |  | UCYN-A1 | |  | UCYN-A2 | |
| --- | --- | --- | --- | --- | --- | --- | --- | --- |
|  | *K* | *P* |  | *K* | *P* |  | *K* | *P* |
| Temperature | **0.567** | **0.001** |  | **0.679** | **0.003** |  | **0.569** | **0.002** |
| Chl *a* | **0.144** | **0.001** |  | **0.464** | **0.023** |  | 0.303 | 0.171 |
| Salinity | **0.228** | **0.001** |  | **0.690** | **0.002** |  | 0.275 | 0.255 |
| PO_4_^3–^ | **0.168** | **0.001** |  | 0.577 | 0.198 |  | 0.224 | 0.385 |
| Si | **0.210** | **0.001** |  | **0.881** | **0.001** |  | 0.321 | 0.147 |
| NO_2_^–^ | 0.107 | 0.476 |  | **0.509** | **0.043** |  | 0.256 | 0.219 |
| NH_4_^+^ | 0.104 | 0.245 |  | **0.782** | **0.002** |  | 0.182 | 0.710 |
| NO_3_^–^ | 0.167 | 0.532 |  | 0.467 | 0.090 |  | 0.286 | 0.541 |

**Table S7** Mantel test showing the relationships between environmental variables and UCYN-A communities. Environmental variables include temperature, salinity, the distance from the sea surface to the bottom (DSB), silicate (SiO_3_^2–^), ammonium (NH^4+^), chlorophyll *a* (Chl *a*), nitrate (NO_3_^–^), nitrite (NO_2_^–^), phosphate (PO_4_^3–^), and the N:P ratio (N/P).

|  | UCYN-A | |  | UCYN-A1 | |  | UCYN-A2 | |
| --- | --- | --- | --- | --- | --- | --- | --- | --- |
|  | *R* | *P* |  | *R* | *P* |  | *R* | *P* |
| Temperature | **0.486** | **0.001** |  | **0.233** | **0.001** |  | **0.515** | **0.001** |
| Salinity | **0.211** | **0.022** |  | **0.197** | **0.012** |  | **0.200** | **0.033** |
| DSB | **0.330** | **0.002** |  | **0.188** | **0.006** |  | **0.307** | **0.004** |
| Si | -0.036 | 0.558 |  | 0.000 | 0.442 |  | -0.060 | 0.647 |
| NH_4_^+^ | 0.109 | 0.163 |  | **0.154** | **0.011** |  | 0.085 | 0.215 |
| N/P | -0.102 | 0.801 |  | 0.003 | 0.433 |  | -0.113 | 0.845 |
| Chl *a* | 0.033 | 0.289 |  | 0.087 | 0.073 |  | 0.049 | 0.267 |
| PO_4_^3–^ | -0.070 | 0.646 |  | 0.053 | 0.148 |  | -0.076 | 0.603 |
| NO_3_^–^ | -0.036 | 0.517 |  | **0.132** | **0.020** |  | -0.046 | 0.543 |
| NO_2_^–^ | -0.067 | 0.725 |  | 0.140 | 0.031 |  | -0.089 | 0.792 |

**Table S8** The topological features of co-occurrence networks for different sublineages of UCYN-A communities

|  | UCYN-A | UCYN-A1 | UCYN-A2 | UCYN-A3 | UCYN-A4 |
| --- | --- | --- | --- | --- | --- |
| Edges^a^ | 382 | 116 | 40 | 9 | 6 |
| Nodes^b^ | 87 | 28 | 34 | 8 | 4 |
| Average degree^c^ | 9 | 8 | 2 | 2 | 3 |
| Connectance^d^ | 0.102 | 0.307 | 0.071 | 0.321 | 1.000 |
| Modularity^e^ | 0.411 | 0.407 | 0.162 | 0.605 | 0.364 |
| Average path length^f^ | 3.203 | 3.174 | 1.475 | 2.723 | 2.607 |
| Clustering coefficient^g^ | 0.572 | 0.591 | 0.796 | 0.259 | 0.462 |
| Average path length ± SD^h^ | 2.52 ± 0.042 | 2.106 ± 0.061 | 3.569 ± 0.4 | 2.181 ± 0.392 | 1.338 ± 0.215 |
| Clustering coefficient ± SD^i^ | 0.212 ± 0.017 | 0.385 ± 0.037 | 0.073 ± 0.048 | 0.131 ± 0.181 | Na |

^a^ Number of edges

^b^ Number of nodes

^c^ Node connectivity; shows the average of the number of connections each node has to another unique node in the network

^d^ Graph density; the intensity of connections among nodes

^e^ Modularity >0.4 indicates that the network has a modular structure. A highly modularized network suggests that there are nodes in the network that are more densely connected with each other than with the rest of the network.

^f^ The length of all the shortest paths from or to the nodes in the network

^g^ Transitivity; measures the probability that the adjacent nodes of a vertex are connected (Barrat, Barthelemy, Pastor-Satorras, & Vespignani, 2004; Newman, 2006; Scott, 2016)

^h^ The average length of all the shortest paths from or to the nodes in the random network

^i^ Transitivity; measures the probability that the adjacent nodes of a vertex are connected in the random network

**References**

Barrat, A., Barthelemy, M., Pastor-Satorras, R., & Vespignani, A. (2004). The architecture of complex weighted networks. *Proc Natl Acad Sci U S A, 101*(11), 3747-3752. doi:10.1073/pnas.0400087101

Newman, M. E. (2006). Modularity and community structure in networks. *Proc Natl Acad Sci U S A, 103*(23), 8577-8582. doi:10.1073/pnas.0601602103

Scott, J. (2016). Social Network Analysis. *Sociology, 22*(1), 109-127. doi:10.1177/0038038588022001007
